# Suprahyoid Muscle Activity and Fatigue During Tongue Elevation Compared With the Head Lift Exercise: A Surface EMG Study

**DOI:** 10.1111/joor.70243

**Published:** 2026-06-12

**Authors:** Tomoki Nanto, Yuta Nakao, Kazuki Eimoto, Yuki Oshima, Shigeo One, Rumiko Maeda, Shumpei Aizawa, Yuka Suetsugi, Takahiro Ono

**Affiliations:** ^1^ Department of Speech‐Language‐Hearing Therapy, Faculty of Rehabilitation Morinomiya University of Medical Sciences Osaka Japan; ^2^ Department of Rehabilitation, Faculty of Allied Health Sciences Yamato University Suita Japan; ^3^ Department of Rehabilitation Hyogo Medical University Hospital Nishinomiya City Hyogo Japan; ^4^ Department of Geriatric Dentistry Osaka Dental University Osaka Japan

**Keywords:** electromyography, head lift exercise, suprahyoid muscles, swallowing rehabilitation, tongue elevation

## Abstract

**Background:**

Strengthening the suprahyoid muscles is fundamental for effective swallowing rehabilitation. Although the head lift exercise is widely employed, it can prove challenging for certain patients. Tongue elevation against the hard palate has been proposed as a feasible alternative. However, its fatigue profile remains underexplored.

**Objective:**

To compare suprahyoid muscle activity and fatigue characteristics during tongue elevation against resistance and the head lift exercise using surface electromyography (sEMG).

**Methods:**

Eighteen healthy adults performed the head lift exercise and maximal isometric tongue elevation task, each sustained for 20 s. Suprahyoid muscle activity was recorded via sEMG and expressed as normalised root mean square (%RMS) and integrated electromyography (%IEMG). Fatigue was assessed using mean frequency (MNF) and median frequency (MDF) and their linear regression slopes over time. Anterior tongue pressure during tongue elevation was measured with a tongue pressure sensor sheet system.

**Results:**

No significant differences emerged between exercises in %RMS or %IEMG. Both tasks exhibited progressive declines in MNF and MDF. Although MNF slopes did not differ significantly, the MDF slope was significantly steeper during tongue elevation than during the head lift exercise (*p* < 0.05), indicating greater fatigue. Maximum tongue pressure was higher at the anterior midline than at the posterior peripheral region.

**Conclusion:**

Tongue elevation may be a feasible alternative or adjunct to the head lift exercise for suprahyoid muscle training, particularly for patients unable to perform the latter task. However, this finding should be interpreted cautiously, given the exploratory design and small sample size of the present study.

## Graphical abstract

Tongue elevation elicited suprahyoid muscle activity comparable to the head lift exercise, despite a greater decline in median frequency over time. Tongue elevation may represent a practical alternative or complementary exercise for suprahyoid muscle training.

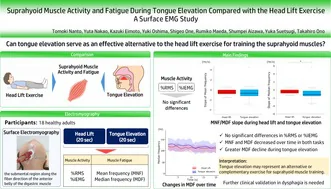

## 1 Introduction

The suprahyoid muscle group—comprising the digastric, mylohyoid, and geniohyoid muscles—contributes to the anterior and superior elevation of the hyoid bone and larynx, which is essential for airway protection (epiglottic inversion and laryngeal closure) and opening of the upper oesophageal sphincter (UES) [1]. Various rehabilitation approaches have been proposed to enhance the strength and endurance of these suprahyoid muscles and thereby improve swallowing function. These include the head lift exercise (Shaker exercise) [2, 3], chin tuck against resistance (CTAR) [4], expiratory muscle strength training (EMST) [5], neuromuscular electrical stimulation (NMES) [6], and related interventions [7]. These methods increase suprahyoid muscle activity and improve swallowing function, as supported by several meta‐analyses [8].

Studies using tongue pressure devices and tongue elevation tasks have also reported increased tongue pressure accompanied by enhanced suprahyoid muscle activation [9]. These findings indicate that tongue elevation facilitates suprahyoid muscle activity and that tongue training may serve as an indirect strengthening method for this muscle group. Furthermore, previous studies have reported no significant differences in electromyographic amplitude between isometric tongue elevation against the hard palate and the head lift exercise, suggesting that tongue elevation may elicit a similar level of suprahyoid muscle activation [10]. Unlike the head lift exercise, which may be challenging for some patients due to limited cervical muscle strength, reduced range of motion, or postural instability, tongue elevation exercises require no cervical movement, impose minimal postural demands, and can be performed without specialised equipment, suggesting high clinical applicability.

From a therapeutic perspective, inducing sufficient muscular effort is essential for promoting neuromuscular adaptation. Previous studies in exercise physiology have demonstrated that higher levels of effort are associated with greater motor unit recruitment [11] and training‐induced adaptations [12]. In addition, fatigue‐related changes in electromyographic frequency have been interpreted as physiological indicators of muscle loading, reflecting alterations in muscle fibre conduction velocity and motor unit behaviour [13]. Electromyographic amplitude indices, such as root mean square (RMS) and integrated electromyography (IEMG), have been used to evaluate suprahyoid muscle activity during tongue elevation exercises [10]. However, these measures alone do not provide complete information about muscle fatigue characteristics. Fatigue‐related indices based on frequency‐domain analysis—which are widely used to assess muscle fatigue [14] have not yet been applied to tongue elevation tasks. Moreover, no previous study has comprehensively compared both amplitude‐ and frequency‐based electromyographic indices between tongue elevation and the head lift exercise. As a result, it remains unclear whether tongue elevation exhibits similar or distinct physiological characteristics to the head lift exercise in terms of both muscle activation and fatigue. Therefore, this study aimed to compare suprahyoid muscle activity during tongue elevation and head lift exercises using surface electromyography (sEMG) and to investigate fatigue‐related indices based on both amplitude and frequency components. We hypothesised that the fatigue characteristics of tongue elevation and head lift exercises are likely to differ.

## 2 Methods

### 2.1 Study Participants

Eighteen healthy adults (8 men and 10 women; mean age, 42.2 ± 16.6 years) participated in this study. None reported a history of speech, language, hearing, or swallowing disorders or any motor impairment of the tongue, lips, jaw, pharynx, or larynx. Additionally, the study participants had no professional background in speech‐language pathology or swallowing rehabilitation, although some were healthcare professionals. Written informed consent was obtained from all participants before participation. The study protocol was approved by the Ethics Committee of Ohmichi‐kai Hospital in Osaka, Japan (approval no. 183).

### 2.2 Sample Size Consideration

Before the main study, a pilot analysis was conducted in 10 participants. Based on this pilot dataset, paired‐samples power analyses (α = 0.05, power = 0.80) were performed using R software (version 4.5.2; R Foundation for Statistical Computing, Vienna, Austria). These analyses indicated that a relatively large sample size would be required to detect statistically significant differences, particularly for frequency‐based fatigue indices. However, previous physiological sEMG studies examining frequency‐domain fatigue indices of the suprahyoid muscles during swallowing‐related or isometric tasks have typically used small sample sizes, often including approximately 10–20 participants [15, 16]. Given the exploratory nature of this study and the discrepancy between pilot‐based power estimates and sample sizes in prior research, the pilot‐derived calculations were not used as the sole basis for formal sample size determination. Instead, the study was designed to evaluate the magnitude of effects detectable with the available sample size. This sensitivity‐based approach allowed for transparent interpretation of both significant and non‐significant findings.

### 2.3 Experimental Procedures

Participants performed a head lift exercise and a maximal‐effort tongue elevation exercise, each sustained for 20 s. Each task was performed once after a brief familiarisation period (approximately 2 s) conducted prior to data collection to ensure task understanding while minimising fatigue. For the head lift exercise, participants were positioned supine on a platform. Following previously described procedures, they were instructed to lift their head while keeping the shoulders in contact with the platform and to raise the head sufficiently to see their toes [3]. They were instructed to keep their jaws closed during both tasks. Data recording began once the head was stably elevated. During the task, verbal cues such as “keep your head lifted so that you can see your toes” were provided as needed to prevent a decline in head position.

For the tongue elevation exercise, participants were instructed to “press the entire tongue firmly against the hard palate” and to maintain maximal effort throughout the 20 s task. Data acquisition began once tongue pressure, measured using a tongue pressure sensor sheet (described below), reached a plateau, defined as a stable waveform showing minimal fluctuation for at least 1–2 s. The order of the exercises was counterbalanced across participants, and a 5 min rest period was provided between tasks [15]. For both exercises, data recording began within 2 s after task onset.

### 2.4 Tongue Pressure Measurement

**FIGURE 1:**
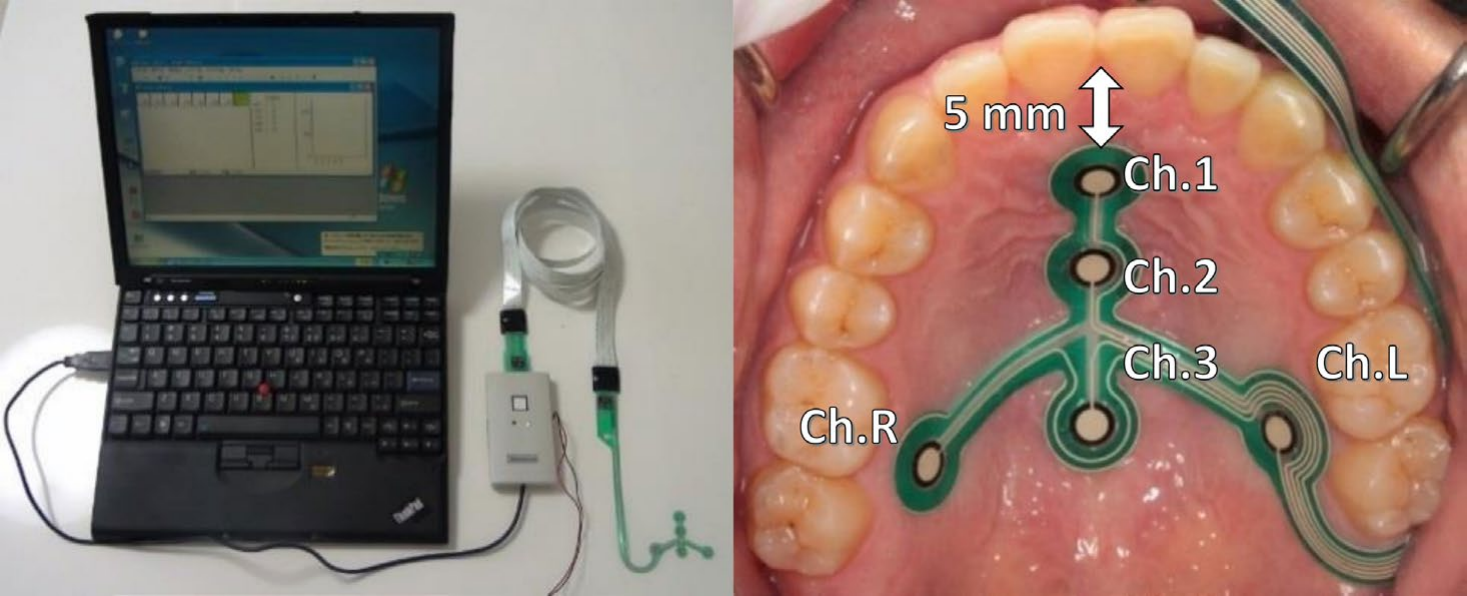
Tongue pressure measurement system. The left panel shows the recording system consisting of a personal computer and a tongue pressure sensor sheet system. The right panel illustrates the placement of the multi‐channel tongue pressure sensor on the hard palate. Channels 1 (Ch. 1), 2 (Ch. 2), and 3 (Ch. 3) were positioned along the midline at 5 mm posterior to the incisive papilla. Channels R (Ch. R) and L (Ch. L) were placed on the right and left peripheral regions of the palate, respectively.

Tongue pressure measurements were included as a secondary exploratory outcome to characterise the spatial distribution and magnitude of tongue–palate contact during the tongue elevation task. Tongue pressure was measured using a tongue pressure sensor sheet system (Swallow Scan; Nitta Corporation, Japan). An ultra‐thin sensor sheet (0.1 mm thick) provided real‐time feedback on the location and magnitude of tongue–palate contact without interfering with tongue elevation. The sensor sheet contained five pressure‐sensitive points at the anterior median (Ch. 1), middle median (Ch. 2), posterior median (Ch. 3), right posterior peripheral (Ch. R), and left posterior peripheral (Ch. L) regions. The sheet was affixed to the hard palate using a denture adhesive (Touch Correct II; Shionogi & Co. Ltd., Japan), with the anterior edge positioned 5 mm posterior to the incisive papilla (Figure 1).

Participants were instructed to elevate the tongue as forcefully as possible while pressing the entire tongue against the hard palate. Real‐time visual feedback from a monitor allowed participants to confirm contact between the tongue and the anterior, middle, and posterior median regions of the hard palate throughout the 20 s task. Tongue pressure signals were displayed at a sampling frequency of 100 Hz, and participants received verbal encouragement to maintain contact with all regions during the task. Tongue pressure data from the 20 s task were analysed to determine the maximum and integrated tongue pressure for each measurement channel. Maximum tongue pressure was defined as the peak pressure value (kPa) achieved during the task, representing the maximal force generation capacity of the tongue, whereas integrated tongue pressure (kPa·s) was calculated as the area under the pressure–time curve, reflecting the cumulative pressure exerted over the duration of the task.

### 2.5 Electromyographic Recording and Signal Processing

**FIGURE 2:**
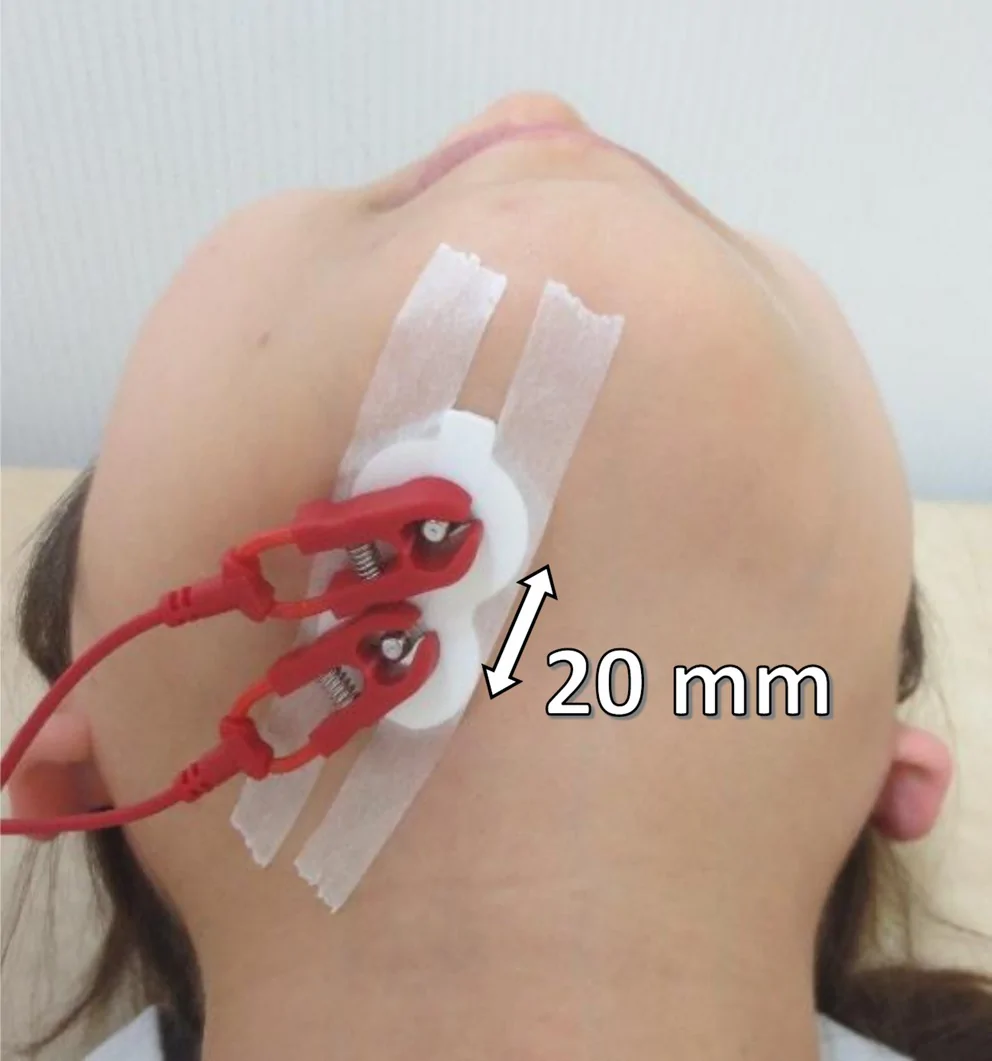
Placement of surface electromyography (EMG) electrodes on the submental region. Bipolar electrodes were positioned along the fibre direction of the anterior belly of the digastric muscle with an inter‐electrode distance of 20 mm.

Suprahyoid muscle activity was recorded using sEMG (Wireless EMG Logger, LP‐WS1046; Logical Product, Fukuoka, Japan). Before electrode placement, the submental skin was cleaned with an alcohol‐soaked cotton swab and prepared with a skin preparation gel (SkinPure; Nihon Kohden, Tokyo, Japan) to ensure stable electrode contact. Bipolar surface electrodes (Duo‐Trode; Myotronics Inc., Kent, WA, USA; inter‐electrode distance, 20 mm) were placed on the submental region along the fibre direction of the anterior belly of the digastric muscle (Figure 2). EMG signals were acquired at a sampling frequency of 1 000 Hz. The recorded signals underwent band‐pass filtering with a 10th‐order Butterworth filter (passband, 10–400 Hz), followed by zero‐phase filtering to prevent phase distortion [17].

Muscle activity was quantified using RMS and IEMG values calculated over the entire 20 s task duration. RMS and IEMG values were normalised to those obtained during the first second of the head lift exercise (defined as 100%), yielding %RMS and %IEMG. This normalisation approach was adopted because obtaining a reliable maximum voluntary contraction (MVC) of the suprahyoid muscles is technically challenging. Identifying tasks that elicit true maximal contraction is not straightforward, and the small, overlapping anatomy of these muscles limits their isolation and assessment [18]. In addition, MVC measures may vary depending on task instructions and participant effort. Therefore, a task‐based normalisation approach was used, with the head lift exercise serving as a standardised and reproducible reference condition widely used in swallowing research. A similar normalisation strategy has also been employed in our previous study [14]. To evaluate muscle fatigue, frequency‐domain analysis was performed on the EMG signals. No single index was prespecified as the primary fatigue outcome; instead, both mean frequency (MNF) and median frequency (MDF) were assessed to enable a complementary and multidimensional assessment of muscle fatigue. MNF and MDF were calculated for each 1 s period throughout the 20 s task. Linear regression analysis was applied to the time series of MNF and MDF values, with the slope of the regression line serving as a fatigue index (MNF slope and MDF slope), based on previous studies [15]. Electromyographic signal processing was performed using R software (version 4.5.2; R Foundation for Statistical Computing, Vienna, Austria).

### 2.6 Statistical Analysis

Data normality for all outcome variables, including electromyographic indices and tongue pressure measures, was assessed using the Shapiro–Wilk test. For electromyographic amplitude indices (%RMS and %IEMG), comparisons between the head lift and tongue elevation tasks were performed using paired *t*‐tests, as normality assumptions were satisfied. For fatigue‐related indices derived from frequency‐domain analysis (MNF slope and MDF slope), normality was assessed using the Shapiro–Wilk test. As these variables did not meet normality assumptions, comparisons between tasks were conducted using the Wilcoxon signed‐rank test for paired samples. To explore potential order effects, additional analyses were conducted for the primary electromyographic outcome measures using repeated‐measures analysis of variance (RM‐ANOVA), with task (head lift vs. tongue elevation) as a within‐subject factor and order (task sequence) as a between‐subject factor. Both main effects of order and task × order interactions were examined.

For tongue pressure variables, different statistical approaches were applied depending on the distributional properties of the data. For maximum tongue pressure, normality assumptions were satisfied across channels. Sphericity was assessed using Mauchly's test, and when violated, Greenhouse–Geisser corrections were applied. One‐way RM‐ANOVA was used to compare maximum tongue pressure across the five measurement channels (anterior, middle, posterior, right peripheral, and left peripheral), followed by post hoc paired *t*‐tests with Holm correction. For integrated tongue pressure, as normality assumptions were not satisfied in some channels, the Friedman test was used as a nonparametric alternative to RM‐ANOVA, with post hoc paired Wilcoxon signed‐rank tests and Holm correction.

Effect sizes were reported as Cohen's dz for paired *t*‐tests, r for Wilcoxon signed‐rank tests, generalised eta squared (ηg^2^) for RM‐ANOVA, and Kendall's W for the Friedman test. All statistical analyses were performed using R software (version 4.5.2; R Foundation for Statistical Computing, Vienna, Austria), and the significance level was set at *p* < 0.05.

## 3 Results

### 3.1 Electromyography

**FIGURE 3:**
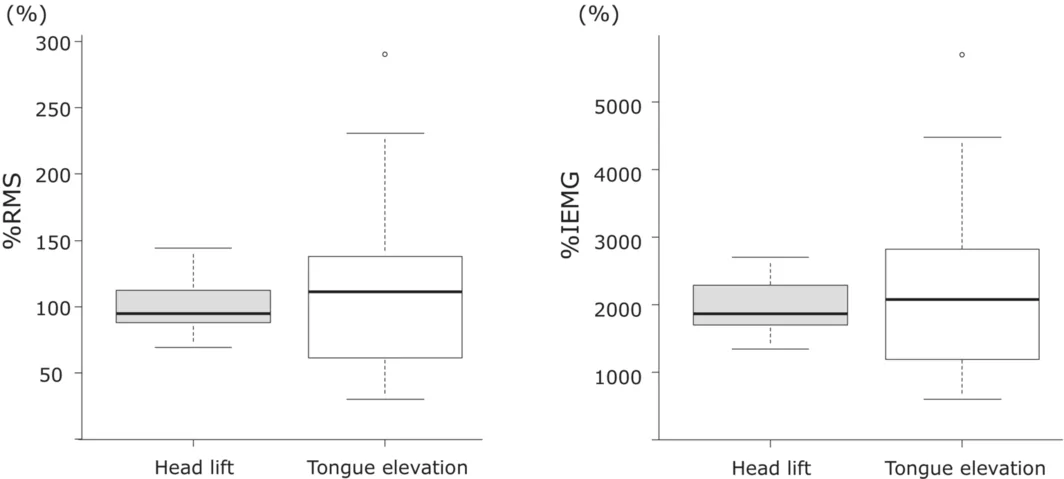
Comparison of muscle activity between head lift and tongue elevation tasks. Normalised electromyographic indices during the head lift and tongue elevation tasks are shown as box plots. The left panel represents the per cent root mean square (%RMS), and the right panel represents the per cent integrated electromyography (%IEMG). No significant differences were observed between the two tasks. Boxes represent the interquartile range, the central line indicates the median, whiskers indicate the range, and outliers are shown in the graph.

Suprahyoid muscle activity results are shown in Figure 3 and Table 1. No significant differences were observed in %RMS or %IEMG over the 20 s task duration between the head lift exercise (%RMS: 99.1% ± 19.0%, %IEMG: 1958.6% ± 377.3%) and the tongue elevation exercise (%RMS: 114.5% ± 64.6%, %IEMG: 2214.3% ± 1260.3%) (%RMS: *p* = 0.375; %IEMG: *p* = 0.446). The effect sizes for these between‐task differences were small (%RMS: Cohen's dz = 0.21; %IEMG: Cohen's dz = 0.18), and the 95% confidence intervals (CIs) for the mean differences included zero (%RMS: −51.6% to 20.3%; %IEMG: −946.6% to 435.1%). The standard deviations (SDs) of %RMS and %IEMG were larger during tongue elevation than during the head lift exercise, indicating greater inter‐individual variability in muscle activation patterns during tongue elevation.

**TABLE 1:** Comparison of suprahyoid muscle activity and fatigue‐related indices between the head lift and tongue elevation exercises.

Variable	Head lift (mean ± SD)	Tongue elevation (mean ± SD)	Test	Statistic	df	*p*	Effect size	95% CI
%RMS	99.1% ± 19.0%	114.5% ± 64.6%	paired t	t = −0.91	df = 17	0.375	dz = 0.21	−51.6% to 20.3%
%IEMG	1958.6% ± 377.3%	2214.3% ± 1260.3%	paired t	t = −0.78	df = 17	0.446	dz = 0.18	−946.6% to 435.1%
MNF slope	−0.48 ± 0.56 Hz/s	−0.60 ± 1.59 Hz/s	Wilcoxon signed‐rank	V = 108	—	0.338	*r* = 0.23	−0.36 to 0.99 Hz/s
MDF slope	−0.43 ± 0.58 Hz/s	−0.89 ± 1.97 Hz/s	Wilcoxon signed‐rank	V = 132	—	**0.045**	*r* = 0.48	0.03 to 1.46 Hz/s

*Note:* Values are presented as mean ± standard deviation. %RMS, normalised root mean square; %IEMG, normalised integrated electromyography; MNF, mean frequency; MDF, median frequency. Effect sizes are reported as Cohen's dz for paired *t*‐tests and r for Wilcoxon signed‐rank tests. 95% confidence intervals (CIs) are provided for the mean or median differences. df is not applicable for nonparametric tests. Bold text indicates *p*‐values < 0.05.

**FIGURE 4:**
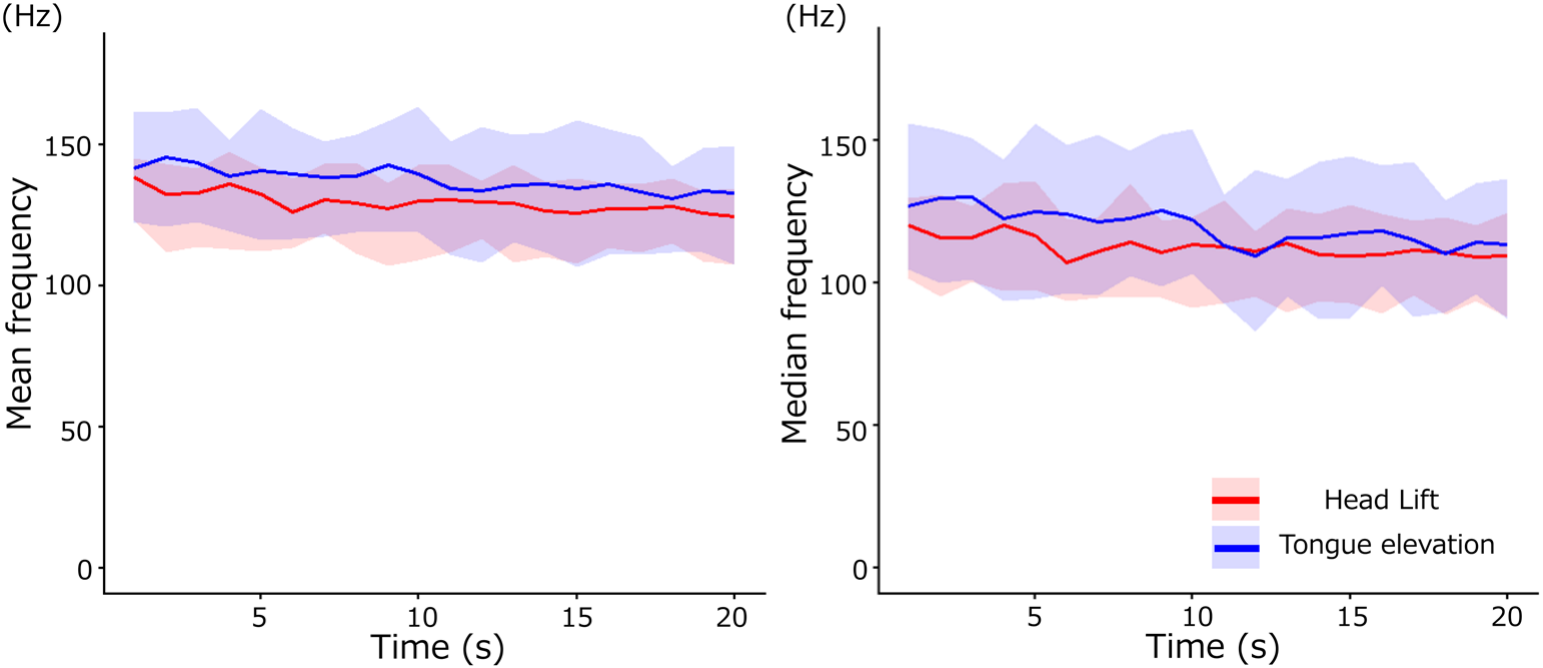
Temporal changes in mean and median frequency during head lift and tongue elevation tasks. The left panel illustrates the time‐dependent changes in mean frequency (MNF), while the right panel illustrates the time‐dependent changes in median frequency (MDF) during the 20 s task. The red line corresponds to the head lift exercise, and the blue line corresponds to tongue elevation against the hard palate. Solid lines represent the mean values across participants, and the shaded regions indicate the interquartile range (IQR), reflecting inter‐individual variability.

**FIGURE 5:**
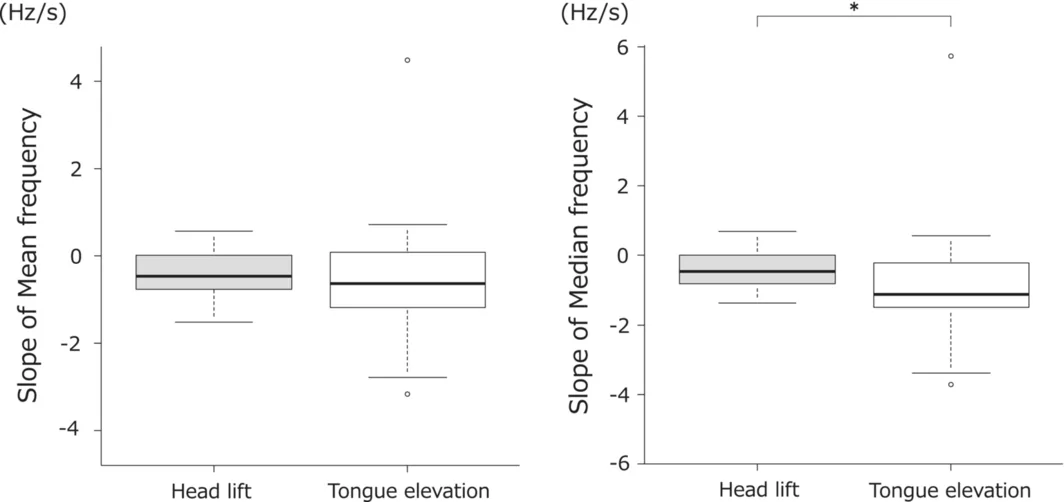
Comparison of frequency slope indices between head lift and tongue elevation tasks. The slopes of the time‐dependent changes in mean frequency (left panel) and median frequency (right panel) during the head lift and tongue elevation tasks are shown. Significant differences are indicated by symbols (*p < 0.05). The slope of median frequency showed a significantly greater decline during the tongue elevation task. Boxes represent the interquartile range, the central line indicates the median, whiskers indicate the range, and outliers are shown in the graph.

The temporal changes in MNF and MDF across the 20 s task are shown in Figure 4. In both tasks, MNF and MDF tended to decrease over time, with variability observed across participants. Regarding fatigue‐related indices from frequency‐domain analysis, no significant difference was found between tasks for the slope of mean frequency (MNF slope) (head lift: −0.48 ± 0.56 Hz/s; tongue elevation: −0.60 ± 1.59 Hz/s; *p* = 0.338) (Figure 5). The effect size for the MNF slope difference was small (*r* = 0.23), and the 95% CI for the median difference included zero (−0.36 to 0.99 Hz/s). In contrast, a significant difference was observed for the slope of median frequency (MDF slope), with a steeper decline during tongue elevation than during the head lift exercise (head lift: −0.43 ± 0.58 Hz/s; tongue elevation: −0.89 ± 1.97 Hz/s; *p* = 0.045) (Figure 5). The effect size indicated a moderate difference (*r* = 0.48), and the 95% CI for the median difference did not include zero (0.03 to 1.46 Hz/s).

No significant main effects of task order were observed for any outcome measures (%RMS: *p* = 0.935; %IEMG: *p* = 0.993; MNF slope: *p* = 0.307; MDF slope: *p* = 0.285). In addition, no significant task‐by‐order interactions were found (%RMS: *p* = 0.540; %IEMG: *p* = 0.551; MNF slope: *p* = 0.099; MDF slope: *p* = 0.137).

### 3.2 Tongue Pressure

**FIGURE 6:**
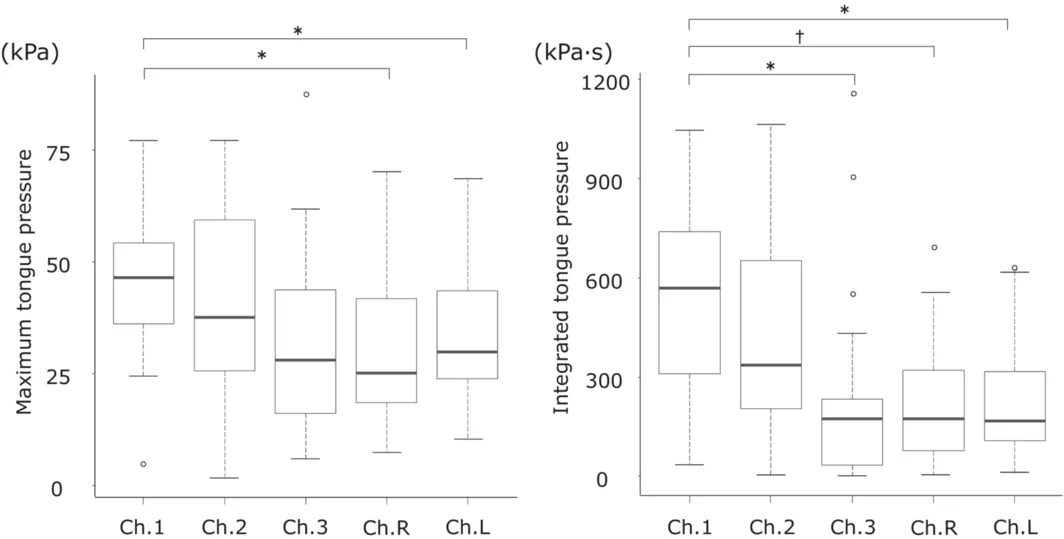
Maximum and integrated tongue pressure by measurement site. Box plots show the maximum tongue pressure (left panel) and integrated tongue pressure (right panel) for each measurement channel (Ch. 1, Ch. 2, Ch. 3, Ch. R, and Ch. L). Significant differences between channels are indicated by symbols (*p < 0.05, † *p* < 0.01). Boxes represent the interquartile range, the central line indicates the median, whiskers indicate the range, and outliers are shown in the graph.

Tongue pressure measurement results are summarised in Figure 6, Tables 2 and 3. Only statistically significant comparisons are shown. Shapiro–Wilk tests indicated that maximum tongue pressure values were normally distributed across all measurement channels. However, Mauchly's test revealed a violation of the sphericity assumption (*p* = 0.016). Therefore, a Greenhouse–Geisser epsilon correction was applied to the one‐way RM‐ANOVA, which demonstrated a significant main effect of measurement channel on maximum tongue pressure (*p* = 0.013). Post hoc pairwise comparisons with Holm correction revealed that maximum tongue pressure at the anterior midline region (Ch. 1: 44.9 ± 16.5 kPa) was significantly greater than at the right posterior peripheral region (Ch. R: 31.3 ± 18.4 kPa; *p* = 0.018) and the left posterior peripheral region (Ch. L: 32.7 ± 14.7 kPa; *p* = 0.039). No other significant differences were observed between channels. For integrated tongue pressure, the Shapiro–Wilk test indicated that normality was not satisfied for the posterior median channel (Ch. 3). Accordingly, a Friedman test was conducted and revealed a significant difference among channels (*p* < 0.001). Subsequent post hoc comparisons using paired Wilcoxon signed‐rank tests with Holm correction showed that integrated tongue pressure at the anterior midline region (Ch. 1: 527.0 ± 295.5 kPa·s) was significantly greater than at the posterior median region (Ch. 3: 257.3 ± 311.8 kPa·s; *p* = 0.036), the right posterior peripheral region (Ch. R: 230.7 ± 194.2 kPa·s; *p* = 0.009), and the left posterior peripheral region (Ch. L: 248.8 ± 191.2 kPa·s; *p* = 0.036).

**TABLE 2:** Statistical analysis of maximum and integrated tongue pressure across palatal measurement channels.

Variable	Test	Statistic	df/corrected df	*p*	Effect size
Maximum tongue pressure	RM‐ANOVA with Greenhouse–Geisser correction	F = 4.02	df = 2.48, 42.20	**0.013**	ηg^2^ = 0.075
Integrated tongue pressure	Friedman test	χ^2^ = 22.4	df = 4	**< 0.001**	Kendall's W = 0.31

*Note:* Values represent results of within‐subject comparisons across measurement channels. RM‐ANOVA, repeated‐measures analysis of variance; GG, Greenhouse–Geisser correction; ηg^2^, generalised eta squared; χ^2^, chi‐square statistic. Kendall's W is reported as the effect size for the Friedman test. df indicates degrees of freedom; corrected df are reported for RM‐ANOVA following Greenhouse–Geisser adjustment. Bold text indicates *p*‐values < 0.05.

**TABLE 3:** Post hoc comparisons of maximum and integrated tongue pressure across palatal measurement channels.

Variable	Comparison	Mean difference	Test	Statistic	df	Holm‐adjusted *p*	Effect size
Maximum tongue pressure	Ch. 1 vs. Ch. R	13.6 kPa	paired t	t = 3.69	17	**0.018**	dz = 0.87
	Ch. 1 vs. Ch. L	12.2 kPa	paired t	t = 3.29	17	**0.039**	dz = 0.78
Integrated tongue pressure	Ch. 1 vs. Ch. 3	269.7 kPa·s	Wilcoxon signed‐rank	V = 152	—	**0.036**	*r* = 0.49
	Ch. 1 vs. Ch. R	296.3 kPa·s	Wilcoxon signed‐rank	V = 162	—	**0.009**	*r* = 0.56
	Ch. 1 vs. Ch. L	278.2 kPa·s	Wilcoxon signed‐rank	V = 152	—	**0.036**	*r* = 0.49

*Note:* Values represent pairwise comparisons between measurement channels. Mean differences are expressed in kPa for maximum tongue pressure and in kPa·s for integrated tongue pressure. Effect sizes are reported as Cohen's dz for paired *t*‐tests and r for Wilcoxon signed‐rank tests. *p*‐values were adjusted using the Holm method for multiple comparisons. df indicates degrees of freedom; df is not applicable for nonparametric tests. Bold text indicates *p*‐values < 0.05.

## 4 Discussion

In this exploratory study, we compared suprahyoid muscle activity and fatigue between the tongue elevation and the head lift tasks. No significant differences were observed in %RMS or %IEMG, although greater variability was noted during tongue elevation. MNF and MDF decreased over time in both tasks, with a greater decline in MDF during tongue elevation. Tongue pressure measurements demonstrated that participants predominantly generated pressure at the anterior midline region during this task, characterising the spatial distribution of tongue–palate contact and providing supporting information for interpreting task performance. However, these findings should be interpreted cautiously, given the exploratory design and limited sample size.

### 4.1 Suprahyoid Muscle Activity and Frequency‐Domain Analysis

The absence of significant differences in %RMS and %IEMG between tongue elevation and head lift may be interpreted in the context of a previous study reporting high suprahyoid muscle activity during tongue elevation [10]. During anterior tongue pressure tasks, increased activity has been reported in the posterior fibres of the genioglossus, mylohyoid, anterior belly of the digastric, medial pterygoid, and intrinsic tongue muscles [19]. These findings suggest that anterior tongue elevation activates suprahyoid muscles. However, because EMG recordings were obtained from the submental region, these results reflect composite regional activity rather than individual muscle activation.

In this study, high tongue pressure was observed at the anterior hard palate during tongue elevation, suggesting sufficient muscle activation. The SDs of %RMS and %IEMG were larger during tongue elevation relative to head lift, suggesting greater inter‐individual variability in tongue contact location and motor strategy. Because head lift uses body weight as a stable load, muscle activity may have been more consistent across participants. Therefore, although no statistically significant differences were observed, these findings should be interpreted cautiously, and no conclusion regarding equivalence between the tasks can be drawn.

When evaluating muscle loading, fatigue‐related indices such as frequency decreases should be considered alongside activity levels [13, 20]. A frequency decline is generally interpreted as an indicator of muscle fatigue, reflecting decreased muscle fibre conduction velocity and altered motor unit firing behaviour [21]. Therefore, fatigue was assessed using frequency‐domain indices. No between‐task difference was found in MNF slope, whereas the MDF slope showed a greater decline during tongue elevation. MDF has been reported to exhibit slightly greater sensitivity to fatigue than MNF, despite its higher variability [13], suggesting that the observed MDF decline may more sensitively reflect fatigue‐related changes during tongue elevation. The greater decline in MDF may reflect differences in motor unit recruitment and fatigue characteristics between tasks. Tongue elevation requires sustained fine motor control and continuous adjustment of tongue–palate contact, potentially increasing variability in motor unit firing and leading to earlier shifts toward lower‐frequency components. This interpretation is supported by evidence that speech‐related musculature exhibits higher and more variable motor unit discharge rates than limb muscles [18].

In contrast, the head lift exercise involves maintaining the weight of the head against gravity in a mechanically constrained isometric task with a relatively stable load, which may promote more consistent motor unit recruitment patterns. Motor unit recruitment during isometric constant‐force contractions is generally characterised by an orderly pattern and a relatively stable motor unit pool, providing more consistent neuromuscular activation compared to dynamic conditions [22]. These differences may contribute to the greater MDF decline observed during tongue elevation. However, because no between‐task difference was observed in MNF and the study was exploratory with a limited sample size, these findings should be interpreted cautiously. The greater MDF decline may reflect task‐specific fatigue characteristics that warrant further investigation.

Following previous methods [15], we performed frequency analysis at 1 s intervals and evaluated fatigue using regression line slopes. To minimise carryover fatigue between tasks, we analysed a short 20 s duration. Nevertheless, decreases in both MNF and MDF were observed in both tasks. Previous studies [15, 16] using similar methods also reported negative regression slopes, indicating muscle fatigue during both head lift and tongue elevation in this study.

The 20 s holding time for the isometric head was based on previous studies. Ferdjallah et al. [16]. recorded surface EMG during sustained supine head lift and evaluated fatigue using frequency analysis, reporting that MDF decreased during sustained holds and that fatigue progressed within 20–60 s. Thus, a 20 s duration is sufficient to detect fatigue‐related frequency changes. Additionally, White et al. [15], compared 20, 40, and 60 s isometric head lift holds in the Shaker exercise and evaluated muscle fatigue using surface EMG frequency analysis. They found that MDF decreased even at 20 s and that fatigue increased with duration. Notably, a 20 s hold was deemed feasible for clinical settings and suitable for quantitative fatigue evaluation.

Collectively, the 20 s head lift duration in this study is supported by previous research showing that muscle fatigue can be evaluated via frequency analysis over this timeframe. This duration balances clinical feasibility and evaluability and was thus adopted. Future studies should examine muscle activity and frequency characteristics over longer durations. However, the greater variability observed during tongue elevation may reflect inter‐individual differences in motor coordination strategies. Unlike the head lift exercise, which imposes a relatively consistent external load through the weight of the head, tongue elevation requires fine motor control and allows multiple movement strategies. Consequently, participants may have differed in how they coordinated tongue elevation and submental muscle recruitment. Additionally, variability in tongue–palate contact patterns and anatomical factors may have contributed to the observed heterogeneity. Tongue pressure and contact patterns are known to be influenced by hard palate morphology and oral cavity structure [23, 24]. Variations in these anatomical features may have affected both tongue–palate contact behaviour and electromyographic activity. Given the exploratory nature and limited sample size, these findings should be interpreted with caution.

### 4.2 Tongue–Palate Contact During Tongue Elevation

Although tongue pressure was not a primary outcome of this study, several findings provided insight into movement patterns during the tongue elevation exercise. Both maximum and integrated tongue pressure were higher at the anterior midline region (Ch. 1) than at the bilateral posterior peripheral regions (Ch. R and Ch. L). This distribution likely reflects the task instructions, which emphasised tongue contact with the hard palate along the anterior to posterior midline. Previous studies have similarly reported higher tongue pressure at the anterior tongue during swallowing and tongue pressure measurements [25, 26]. Accordingly, instructions to elevate the tongue along the anterior–posterior direction likely contributed to the higher pressure observed at the anterior palate and tongue regions.

For integrated tongue pressure, a significant difference was observed between the anterior (Ch. 1) and posterior midline regions (Ch. 3), with numerous outliers in the posterior region, indicating substantial inter‐individual variability. Tongue pressure is influenced by muscle activity and by anatomical factors such as hard palate morphology and oral cavity volume [23, 24]. In this study, greater variability in posterior and peripheral regions may reflect individual differences in tongue–palate contact patterns.

Furthermore, these tongue–palate contact patterns are likely associated with differences in muscle activation and fatigue characteristics. Unlike the head lift exercise, tongue elevation requires coordinated activation of multiple muscle groups, including the suprahyoid and extrinsic tongue muscles [19]. This sustained fine motor control demand may alter motor unit firing and conduction velocity, contributing to the greater MDF decline during tongue elevation.

Variability in tongue–palate contact patterns may influence fatigue characteristics through differences in muscle recruitment. Although tongue pressure was not a primary outcome, these findings may help interpret the electromyographic variability and fatigue‐related changes observed during tongue elevation. Future studies should further investigate tongue–palate contact patterns, particularly in individuals with impaired oral motor function.

### 4.3 Clinical Implications

The head lift exercise is widely implemented worldwide, and its effectiveness for improving swallowing function has been reported [2]. Additionally, CTAR was developed to selectively strengthen the suprahyoid muscles without inducing sternocleidomastoid muscle fatigue and to enable performance even in patients for whom head lifting is difficult [27]. However, CTAR may be difficult for physically frail patients, such as those with neurological disorders after stroke, who may have reduced upper‐limb strength, impaired hand function, and limited joint range of motion [28]. Moreover, CTAR may not be feasible in patients after neck surgery or tracheostomy, or in those with restricted joint range of motion. In such cases, training methods are needed that can elicit suprahyoid muscle contraction without requiring neck flexion and without stretching or compressing the anterior cervical soft tissues. Furthermore, in rehabilitation settings, specialised devices such as tongue pressure measurement systems are often difficult to implement as self‐training or home‐based tasks. Therefore, programs that can be performed without special equipment and with minimal tools are required [29]. The tongue elevation exercise does not require neck movement or special devices, which may be clinically advantageous. Although substantial inter‐individual variability was observed, tongue elevation may represent an alternative or complementary approach for suprahyoid muscle training, particularly in individuals who have difficulty performing the head lift exercise. Further studies are needed to determine its clinical effectiveness and applicability in patients with dysphagia.

### 4.4 Limitations

This study has several limitations. Participants were limited to healthy adults; therefore, the findings may not be generalisable to patients with dysphagia. In addition, measurements were performed only once, long‐term training effects were not evaluated, and surface EMG recordings reflected composite rather than individual suprahyoid muscle activity.

Additionally, RMS and IEMG were normalised to the first second of the head lift exercise as a reference condition. Because this task‐based approach relies on a single reference task, it may limit the interpretation of between‐task comparisons. Future studies should consider alternative normalisation methods, such as MVC‐based or task‐specific normalisation. To account for fatigue, a relatively short duration of 20 s was used. Future studies should consider longer durations, e.g., approximately 1 min, as in previous studies [15, 16]. Although effect sizes were reported, the sample size was limited, and smaller effects may not have been detected. Therefore, non‐significant findings should be interpreted alongside effect sizes and CIs rather than as evidence of no effect. In addition, detailed analysis of tongue–palate contact patterns and their relationship with muscle activation was limited. Future studies should incorporate combined analyses of tongue pressure, electromyographic, and kinematic indices, including patients with dysphagia and training interventions.

## 5 Conclusions

Tongue elevation was associated with suprahyoid muscle activation that did not significantly differ from that observed during the head lift exercise, as reflected by amplitude‐based electromyographic indices. Frequency‐domain analysis revealed fatigue‐related decreases in both exercises, with a significantly greater decline in MDF during tongue elevation. However, given inter‐individual variability and the limited sample size, these findings should be interpreted with caution. Given its minimal postural demands and lack of required equipment, tongue elevation may represent an alternative or complementary exercise for suprahyoid muscle training, particularly in individuals who have difficulty performing the head lift exercise. Further studies are needed to determine its clinical effectiveness and applicability in patients with dysphagia.

## Author Contributions

**Yuta Nakao:** conceptualization, methodology, formal analysis, writing – review and editing, supervision. **Yuki Oshima:** conceptualization, methodology, formal analysis, writing – review and editing. **Takahiro Ono:** conceptualization, methodology, writing – review and editing, supervision. **Yuka Suetsugi:** formal analysis, writing – original draft, writing – review and editing. **Tomoki Nanto:** conceptualization, methodology, project administration, data curation, investigation, formal analysis, writing – original draft, writing – review and editing. **Kazuki Eimoto:** conceptualization, methodology, formal analysis, writing – review and editing. **Rumiko Maeda:** writing – original draft, writing – review and editing. **Shigeo One:** writing – original draft, writing – review and editing. **Shumpei Aizawa:** writing – original draft, writing – review and editing.

## Ethics Statement

The study protocol was approved by the Ethics Committee of Ohmichi‐kai Hospital in Osaka, Japan (approval no. 183).

## Consent

Written informed consent was obtained from all participants before participation.

## Conflicts of Interest

The authors declare no conflicts of interest.

## Data Availability

The data that support the findings of this study are available on request from the corresponding author. The data are not publicly available due to privacy or ethical restrictions.
